# Analysis of Deformation, the Stressed State and Fracture Predictions for Cogging Shafts with Convex Anvils

**DOI:** 10.3390/ma14113113

**Published:** 2021-06-06

**Authors:** Marcin Kukuryk

**Affiliations:** Faculty of Mechanical Engineering and Computer Science, Czestochowa University of Technology, 42-200 Czestochowa, Poland; kukurykm@itm.pcz.pl

**Keywords:** cogging, convex anvils, FEM, strain, stress, damage, shaped anvils

## Abstract

In this article, a new manner of cogging a forging (type: shaft), consisting in the application of a two-stage process composed of preliminary shaping in convex anvils, and also principal forging in flat or shaped anvils, is presented. A new manner of forging brought about the formation of favorable conditions for achieving the maximum values of the effective strain in the central part of a forging, accompanied by a simultaneous absence of tensile stresses, which was exerting a favorable influence upon reforging the axial zone of an ingot. What was determined, was the effective geometric shapes of convex anvils; the efficiency of different technological parameters in the case of the intensity of reforging the axial zone of an ingot was analyzed as well. The investigations were complemented by means of predicting the formation of ductile fractures in the course of forging with the application of three different ductile fracture criteria. The comparison of theoretical and experimental outcomes of investigations indicates a good level of being commensurate.

## 1. Introduction

The objective of the new forging technologies is to achieve forgings which can boast a very high quality, and that means products having the required shape and dimensional tolerance, and also to achieve such properties and structures of a material which are the most favorable in the aspect of the material in the question’s intended application and functional properties of a product [1,2]. Streamlining the construction of machines and devices, which are more and more frequently required to work reliably and without breakdowns at elevated temperatures, and also to be well resistant to a corrosive impact of the environment, requires the application of austenitic stainless steels, the principal components of which are nickel and chromium [3,4].

The issue of selecting the best possible technological process of forging austenitic steels for the purpose of achieving forgings, which can boast high mechanical properties and which are free of internal defects, constitutes an extremely significant problem [5,6]. From a technological point of view, which is particularly significant, is the state of strain and stress in the axial zone of a forged material, determined, first and foremost, by the shape of applied anvils, and also by the values of technological parameters. In this area of an ingot, the largest clusters of defects of the following types: micro-shrinkages, voids, and internal cracks, which, in the course of conducting the forging process, ought to undergo significant reduction or be eliminated altogether, are observed [7,8,9].

The issue of the deformability of austenitic stainless steels is a very significant problem in the processes of thermal-mechanical shaping of cogged forgings, and the process of the forging those steels may be, to a significant degree, rendered more difficult by a low level of plastic properties and rapid strengthening of the forgings in question. Nevertheless, in the case of a certain combination of strain, strain rate, and temperature, a good level of deformability is possible to be achieved in the case of the investigated austenitic steel. That fact is assigned to the phenomena of dynamic recrystallization (DRX) and dynamic recovery (DRV), which change the formed microstructure, together with the applied combination of thermal-mechanical parameters. The forming microstructure is closely connected with mechanical properties of the following types: strength, ductility, and fracture toughness [10,11].

Determining the boundary deformability of a material in the forging processes is a significant issue from the point of view of designing the best possible technologies. One of the directions of investigations into that problem are attempts to determine the properties of the austenitic steel subjected to deformation, the best possible shapes of anvils, and also the parameters of the cogging process, which do not bring about the loss of cohesion by a material and the formation of ductile fractures. The outcome of the conducted investigations demonstrates a significant influence exerted by state of stress, strain rate, temperature, the parameters of microstructures, and also the conditions of friction on the contact surface between a material and a tool, upon the boundary value of the effective strain, after exceeding which material cracking is observed [12,13]. As the outcome of investigations into that issue, numerous criteria relevant to the formation of cracks using different analytical correlations based upon the values of stresses, integrated by means of material strain, were developed [14,15]. As the outcome of a preliminary verification of different criteria of crack formation, for predicting the loss of cohesion by the austenitic steel subjected to deformation in the cogging process, three criteria: the normalized Cockcroft–Latham criterion, Oyane et al. criterion, and Brozzo et al. criterion, were applied.

Metal flow, the continuity of internal construction, and the structure and properties of a forged material are determined by the properties of an initial billet, and also, within the scope of forging technologies, are dependent upon the shape and the dimensions of forging tools, and also upon applied technological parameters [16,17]. Achieving qualitative objectives in the course of cogging austenitic steels is possible exclusively in conditions guaranteeing a favorable thermal-mechanical state, the properties of which include the presence of compression stresses in a deformation valley, in the case of the greatest possible limiting of tensile stresses zones [18,19].

In order to maintain, throughout an entire volume of a forging, a comparatively small gradient of mechanical properties, in the case of simultaneously maintaining high values of them, the forging process ought to be conducted in a manner permitting the axial zone of an ingot to reach the required forging ratio [20,21]. The same forging ratio can be achieved with the application of flat or shaped anvils, but in the case of the application of a different number of passes, which is connected to change in the costs of the production process. 

The objective of this paper was to analyze a new manner of cogging a forging (type: shaft) made of the X5CrNi18-10 austenitic stainless steel, consisting in the application of a two-stage process: a preliminary shaping of an ingot in convex anvils, and also principal forging conducted in flat or shaped anvils. The outcomes of the investigations into the distribution of the effective strain, effective stress, mean stresses, temperature, stress triaxiality, and also predicting crack formation in the course of plastic shaping in three original special convex anvils, and also in flat and V-shaped anvils, are presented.

## 2. Materials and Methods

### 2.1. Finite Element Model Analysis for Deformation and Heat Transfer

In this study, the thermal-mechanical finite element model is used. The solution is based upon a thermo-mechanical approach, combined with solving the Fourier equation for non-stationary heat flows. According to the variational principle, the basic equation for the finite-element formulation is expressed as [22,23]:

(1) $$\delta \prod =\int_{V}\bar{\sigma }\delta \dot{\bar{\varepsilon }}dV+\int_{V}K{\dot{\varepsilon }}_{V}\delta {\dot{\varepsilon }}_{V}dV-\int_{S}F_{i}\delta u_{i}dS=0,$$

where $\bar{\sigma }$ is the equivalent stress, ${\dot{\bar{\varepsilon }}}_{}$ is the equivalent strain rate, ${\dot{\varepsilon }}_{V}$ is the volumetric strain rate, *F_i_* represents surface tractions, *u_i_* is the velocity field, *S* is the surface, *V* is the volume of a sample, *K* is a penalty constant (*K* = 10^6^).

The analysis of heat-transfer can be obtained by solving the energy balance equation, expressed by [24]:

(2) $$k\nabla^{2}T+\dot{q}-\rho c_{}\dot{T}=0,$$

where *c* denotes specific heat, *k* denotes thermal conductivity, *T* is the temperature, *ρ* denotes density, $\dot{q}$ is the heat-generation rate, $k\nabla^{2}T$ is the heat-transfer rate, and $\rho c\dot{T}$ is the heat-generation rate. The heat generation rate in the deformed body is induced by plastic deformation and is given by:

(3) $$\dot{q}=\alpha \bar{\sigma }\dot{\bar{\varepsilon }},$$

where *α* represents the fraction of mechanical energy converted into heat (assumed to be 0.9).

The energy equation, Equation (2), can be rewritten using the weighted residual method as:

(4) $$\int_{V}kT_{,i}\delta T_{,i}dV+\int_{V}\rho c\dot{T}\delta TdV-\int_{V}\alpha \bar{\sigma }\dot{\bar{\varepsilon }}\delta TdV-\int_{S_{q}}q_{n}\delta TdS=0,$$

where *q_n_* is the heat flux normal to the boundary surface. The temperature distribution of the forgings can be obtained readily by solving the energy balance equation, Equation (4). 

The frictional boundary condition is given by the vector form [25]:

(5) $$f=-m\tau \left[\frac{2}{\pi }arc\;tg\left(\frac{\left|u_{s}\right|}{u_{0}}\right)\right]\frac{u_{s}}{\left|u_{s}\right|},$$

where *τ* is the local flow stress in shear, *m* is the friction factor, *u_s_* is the velocity vector of the workpiece relative to the anvil, *u_o_* is a very small positive number (10^−5^) compared to |*u_s_*|. 

Equation (4) as well as the boundary conditions for any arbitrary variation of temperature, *δT*. The finite element method is then adapted to solve Equations (1) and (4) which define the thermo-mechanical state of a body in the thermodynamic process.

The rigid-viscoplastic thermal-mechanical finite-element method was used in the simulation of metal flow and heat transfer during the cogging process. A three-dimensional model simulation was performed with Deform 3D software (V12, Scientific Forming Technologies Corporation, OH, USA). Workpiece was modeled as a free plastic object and it was meshed with about 100,000 tetrahedral elements. In the cogging process of modeling, the contact conditions are constantly updated, reflecting the movement of the anvils and the deformation of the material, which allows to simulate the slip between the anvils and the material of the processing billet. The contact between the anvils and the workpiece is modeled by the shear friction model (Equation (5)). This states that the friction is a function of the shear yield stress of the deforming body. The friction stress *f* on the interface of the billet and the anvils can be expressed approximately as an arctangent function of its relative sliding velocity *u_s_*. The contact pressure can be calculated to an approximate value using the correlation of the surface gap and the penalty constant (the penalty method). Computation is carried out until the complete bonding of the two surfaces is achieved. In the processes of forging ingots, most lubrication is not used. In order to maintain similar conditions in the performed compression tests to the conditions prevailing in the experiment, the investigations were performed without lubrication. According to the experimental setup, in numerical simulation, the shear friction factor between the workpiece and anvils equal to 0.7 was chosen in order to reproduce the friction conditions between anvils and the workpiece. In forging experiments, the friction factor was determined from additional ring compression tests. 

### 2.2. Investigated Material and Theoretical Procedures 

**Figure 1:**
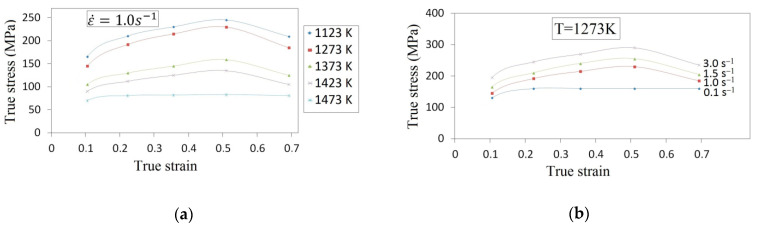
Flow curve plots for specimens deformed at (**a**) constant strain rate of 1.0 s^−1^ and different deformation temperatures and at (**b**) different strain rates and a constant temperature of 1273 K.

The chemical composition of the X5CrNi18-10 stainless steel was as follows (wt. %): 0.07 C, 0.80 Si, 1.55 Mn, 18.05 Cr, 10.15 Ni, 0.025 P, 0.020 S, and Fe in balance. The cylindrical specimens of *φ*30 mm × 30 mm were prepared from the as-cast billet. The investigations encompassed the compression of cylindrical specimens on a Japanese cam plastometer (type: MAEKAWA, Tokyo, Japan). The specimen was subjected to deformation at different temperatures, ranging from 1123 K up to 1473 K, for the following scope of true strains: 0.105–0.693 and for the following strain rates: 0.10–3.0 s^−1^. All the specimens were heated up to the temperature of 1473 K with a heating rate of 30 K/s, homogenized at that temperature for 160 s, cooled at 10 K/s to different temperatures, held for 30 s and deformed at constant strain rates. All the specimens were subjected to deformation until reaching a true strain of 0.693. Thermocouples were welded on the surface of a compression sample to measure temperature. In Figure 1, typical flow curves of the X5CrNi18-10 compressed steel in different conditions of heat deformation are presented. The stresses increase until the peak stress is reached, and afterwards decrease until the intermediate values between the yield stress and the peak stress are reached. This is a typical character of dynamic recrystallization [26]. It is possible to observe that the shape of flow curves is, prevalently, dependent upon the temperature and upon strain rate. The peak stress is increasing as temperature is decreasing (Figure 1a) and strain rate is increasing (Figure 1b). At an initial stage of deformation, the flow stress increases dramatically, and afterwards slowly; the latter indicates that the work-hardening decreases as strain increases. The curves described are valid for 1473 K and 1.0 s^−1^, as well as for 1273 K and 0.1 s^−1^, it is observed that, for true strains exceeding 0.2, the flow curves are nearly stable. The flow curves exhibit a broad stress peak. For a larger value of strain rates, continuous flow softening is observed after reaching a peak stress, which may as well be attributed to the dynamic recrystallization (DRX) [27].

**Figure 2:**
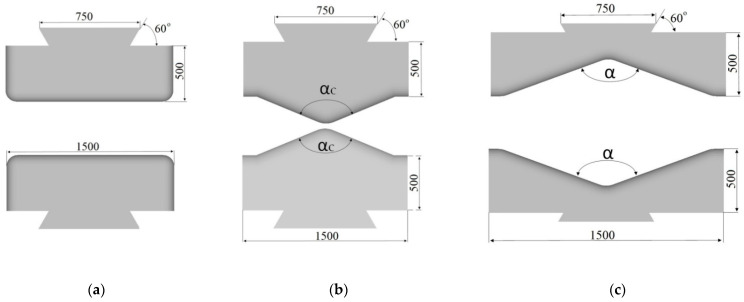
Shape of anvils: (**a**) flat, (**b**) convex (angle: *α_c_* = 135°, 150° and 165°), and (**c**) V-shaped (*α* = 140°), the numbers in the figure in mm.

In numerical calculations, the following ingot dimensions (after eliminating convergences) were assumed: the diameter of *φ*800 mm and the length of 2000 mm; the ingot was made of the X5CrNi18-10 stainless steel. As an initial temperature of an initial billet, the following values were assumed: 1423 K, anvils 523 K, and that means the temperature of heating them in industrial conditions. In order to achieve a good reforging of the axial zone of an ingot, a new effective approach, consisting in the application of convex anvils for the purpose of preliminary forging, was applied. Principal forging was conducted in flat anvils in eight consecutive passes with 90° rotating of the billet, or in V-shaped anvils in four or six passes, determined by the applied reduction ratio (the reduction ratio, *ε_c_*, is defined as ln*H*_1_*/H*, where *H* and *H*_1_ are the height of the initial and the deformed forging (Figure 4), respectively). In the cogging process, the pressing speed of anvil was 20 mm/s. The collation of the applied anvils is presented in Figure 2.

Operating temperature at the cogging process for the investigated austenitic steel consists of heat exchange between anvils, workpiece, and environment, as well as the thermal effect of mechanical energy transferred into heat. Heat transfer is carried out by convective and radiative exchange with the environment from the free surface of a workpiece. A heat convection coefficient to the environment equal to 20 W/(m^2^K) and the emissivity 0.7 N/(s mm K^4^) were assumed in order to reproduce the thermal gradient due to the deformation material cooling time [28]. The characteristics of the properties such as: density, specific heat, and thermal conductivity, were assumed upon the basis of experimental data, and set as the functions of temperature. The value of thermal conductivity coefficient was determined by experimental means. In a round specimen (*φ*80 × 100 mm), six holes were made (diameter: *φ*1.2 mm), at a homogeneous distance from a frontal plane of a specimen (every 15 mm). In these holes, the sheathed thermoelements (K-type), the diameter of which is 1.0 mm, were made and the lateral surface of cylindrical specimens was insulated. The distribution of temperature in the heated specimen was calculated with the application of the finite element method, whereas the boundary condition on a frontal plane was assumed upon the basis of measurements by experimental means. The achieved outcomes of the experimental investigations were approximated with the application of the following analytical correlations in the function of temperature:

— thermal conductivity coefficient *λ* [W m^−1^ K^−1^]
  (6) $$\lambda =-0.00322\cdot {\bar{T}}^{2}+19.45268\cdot \bar{T}+8.2003\;\mathrm{for}\;T\;\geq \;293\;K,$$
— specific heat *c_p_* [J kg^−1^ K^−1^]
  (7) $$c_{p}=0.05180\cdot {\bar{T}}^{2}+197.7733\cdot \bar{T}+433.05189\;\mathrm{for}\;T\;\geq \;293\;K,$$
— density *ρ* [kg m^−3^]
  (8) $$\rho =-0.07804\cdot {\bar{T}}^{2}-\;329.58862\cdot \bar{T}+8010.76\;\mathrm{for}\;T\;\geq \;293\;K,$$
  where $\bar{T}=\frac{T[K]}{1000}$.

### 2.3. Considered Ductile Fracture Criteria

Determining the boundary deformability of a material in the cogging process is a significant issue from the point of view of designing a technological process. As the outcome of investigations into this issue, numerous criteria of the formation of ductile fractures were developed. After a preliminary verification of different ductile fracture criteria for predicting crack formation in the course of plastic shaping of ingots made of the X5CrNi18-10 steel, three criteria: the normalized Cockcroft–Latham criterion, Oyane et al. criterion, and Brozzo et al. criterion were assumed. In the models, it was assumed that reaching the maximum possible level of plasticity and formation of a fracture in a material subjected to deformation will be initiated when the determined value for particular criteria exceeds a certain determined boundary value (*C*_1_, *C*_2_, and *C*_3_).

It is assumed in the model proposed by Cockroft and Latham [29] that the principal tensile stress is the most important parameter for the initiation of discontinuities. It is assumed that the formation of a discontinuity in the material will be initiated, when a certain limiting criterion value representing the ductile fracture has been exceeded. The damage was calculated by the following damage model:

(9) $$\int_{0}^{{\bar{\varepsilon }}_{f}{}_{}}\frac{\sigma_{1}}{\bar{\sigma }}d\bar{\varepsilon }=C_{1},$$

where *σ*_1_ is the maximum principal stress, $\bar{\sigma }$ is the effective stress, $\bar{\varepsilon }$ is the effective strain, and ${\bar{\varepsilon }}_{f}$ is the effective strain of fracture.

The limiting value of the normalized Cockcroft–Latham fracture criterion was determined for the X5CrNi18-10 steel with the application of a comparative method based upon the notch uniaxial tensile test. The limiting normalized Cockcroft–Latham fracture criterion values, as determined by the tensile test for the temperatures examined and assumed strain rates, amounted to *C*_1_ = 0.685.

Oyane et al. [30] proposed a fracture model upon the basis of the proportion of mean stress (*σ_m_*) to the effective stress $\left(\bar{\sigma }\right)$:

(10) $$\int_{0}^{{\bar{\varepsilon }}_{f}}\left[1+\frac{1}{a}\frac{\sigma_{m}}{\bar{\sigma }}\right]d\bar{\varepsilon }=C_{2}$$

In Equation (10), *a* is a parameter scaling the influence exerted by mean stress upon the boundary deformability of a metal. 

In the model of Brozzo et al. [31], the modification of the Cockcroft–Latham fracture criterion was performed; that modification consisted in taking under consideration the influence exerted by maximum tensile stresses (*σ*_1_), and also by mean stress (*σ_m_*), upon the boundary strain of a material in the form of the correlation:

(11) $$\int_{0}^{{\bar{\varepsilon }}_{f}{}_{}}\frac{2\sigma_{1}}{3(\sigma_{1}-\sigma_{m})}d\bar{\varepsilon }=C_{3}$$

For determining the boundary values for the criteria developed by Oyane et al. and by Brozzo et al., it was attempted to conduct a uniaxial compression of cylindrical specimens made of the X5CrNi18-10 steel, the diameter of which was *φ*30.0 mm, and the height of which was varied. The state of stress and strain for compressed specimens was determined with the application of the finite element method, in the case of the application of the DEFORM-3D software. The calculation was conducted for specimens having the slenderness ratio of 0.50–1.50, for four temperatures: 1273, 1323, 1373, and 1423 K, and also variable values of friction factor (0.05–0.70). The values of friction factor assumed for the purpose of calculation were as well determined with the application of the ring compression method. The final height of compression meant the height of the specimen at the moment of observing cracks on a free surface (midway through the specimen height). Having at disposal detailed data on the state of stress and strain, achieved with the application of the finite element method, the following material constants *C*_2_ = 0.753 (Equation (10)) and *C*_3_ = 0.872 (Equation (11)) were determined.

## 3. Results and Discussion

### 3.1. Investigation of Cogging Processes with Convex Anvils

**Figure 3:**
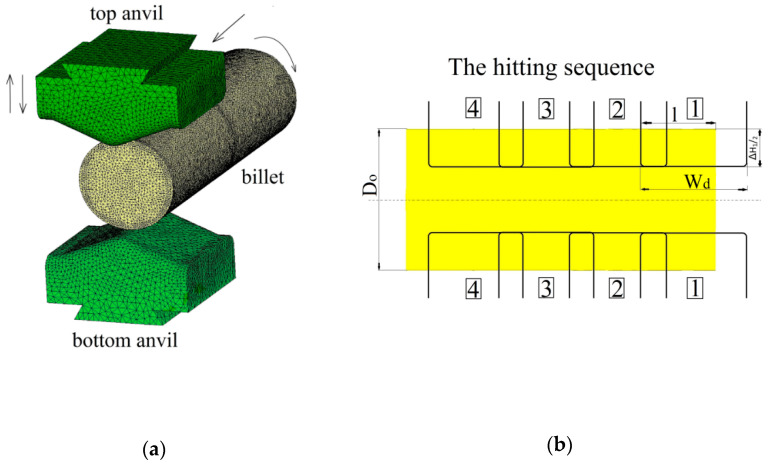

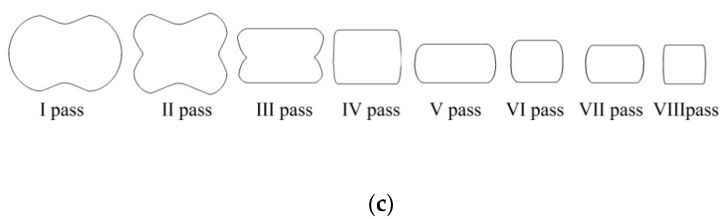
Schematic illustration of the cogging process: (**a**) finite element model for the forging experiment, (**b**) the hitting sequence on the ingot length (l/w_d_ = 0.70; l/D_0_ = 0.75), and (**c**) pass sequence for flat anvils (90° rotation).

**Figure 4:**
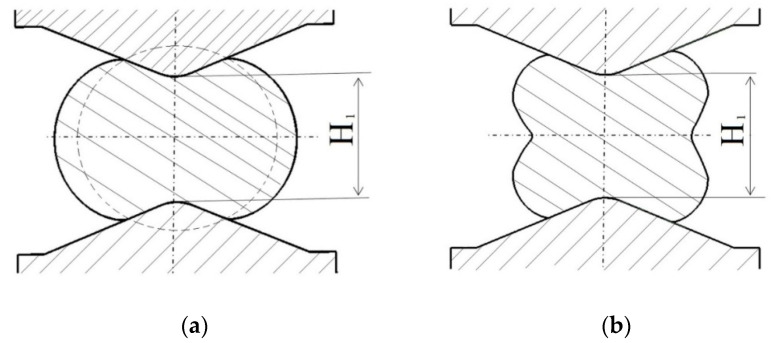
Order of forging the workpiece with convex anvils (*α_c_* = 135°, 150°, and 165°), (**a**) after the first pass, (**b**) after the second pass.

**Figure 5:**
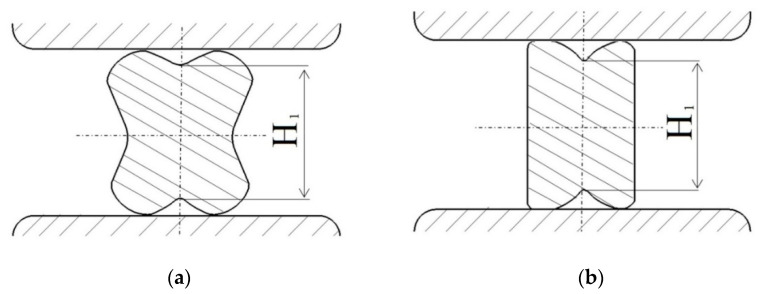
Order of forging the workpiece with a concave cross-section with flat anvils (**a**) after the first pass, (**b**) after the second pass.

**Figure 6:**
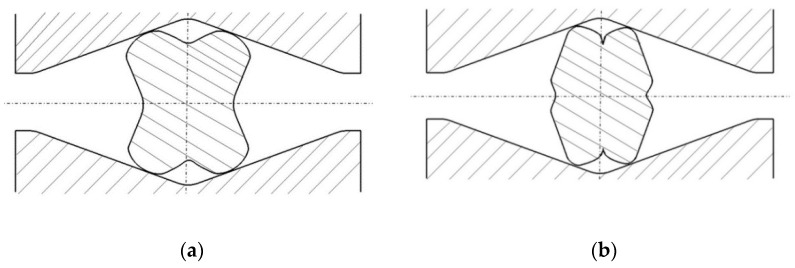
Order of forging the workpiece with a concave cross-section with V-shaped anvils (**a**) after the first pass, (**b**) after the second pass.

In this article, the achieved distributions of the effective strain, the effective stress, mean stresses, and temperature in the cogging process of the X5CrNi18-10 stainless steel in hot forging conditions in three original convex anvils for the purpose of preliminary shaping were analyzed. The further stage of forging was conducted in flat or V-shaped anvils having the following angle: 140° × 140°. Geometric models of anvils and a material subjected to deformation is presented in Figure 3a, the diagram of particular deformations on the length of an ingot is presented in Figure 3b, and the diagram of conducting particular passes in Figure 3c. In the case of convex anvils, two passes with interoperational 90° rotating (Figure 4) were assumed. Further, two passes in the course of forging in flat anvils concerned eliminating the formed concavities after convex anvils (Figure 5). The remaining ones were treated as a series of cyclically repeating passes conducted on flat or V-shaped anvils with interoperational 90° rotating. The process of eliminating concavities, and also forging in V-shaped anvils, is presented in Figure 6. In shaped anvils, retaining the same forging ratio, the forging process was conducted in 4 passes (for the smallest concavity *ε_c_* = 0.22, two more passes were conducted). The forging ratio is defined as *A*_0_/*A*, where *A_0_* is the original forging cross-section area before cogging and *A* is the final forging cross-section area after the cogging process. The schedule of the cogging process, together with the dimensions of the cross-section after particular passes, is presented in Table 1. 

The specific character of the forging process in convex anvils brings about the fact that local strains on the section of a material subjected to deformation after the first pass (Figure 4a) were diversified, nevertheless, they guaranteed a good plastic forming in the limited workability zone. In the area situated adjacently to the surface of anvils, it was observed that there were high values of the effective strain, whereas the lateral zones of forging were the area of those values being smaller. The application of the second pass in convex anvils (Figure 4b) brought about significant changes in the kinematics of metal flow and a significant increase in homogeneous distribution of the effective strain. For that very reason, for conducting the forging process, two passes in convex anvils were applied. 

### 3.2. Influence of the Shape of Convex Anvils and Reduction Ratio on the Effective Strain and the Stressed State of the Workpiece 

**Figure 7:**
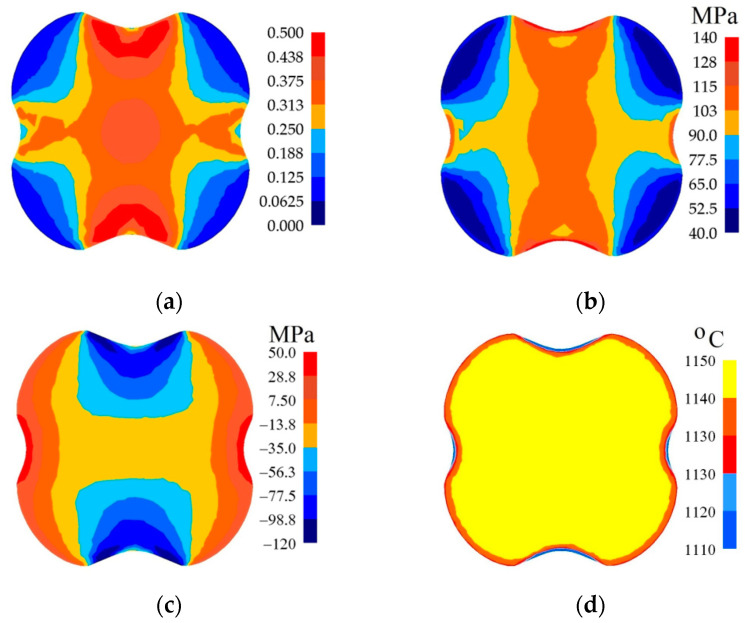
Distribution of the effective strain (**a**), effective stress (**b**), mean stresses (**c**), and temperature (**d**) on the cross-section of the workpiece in the course of forging in convex anvils after the second pass (*α_c_* = 135°, *ε_c_* = 0.22).

**Figure 8:**
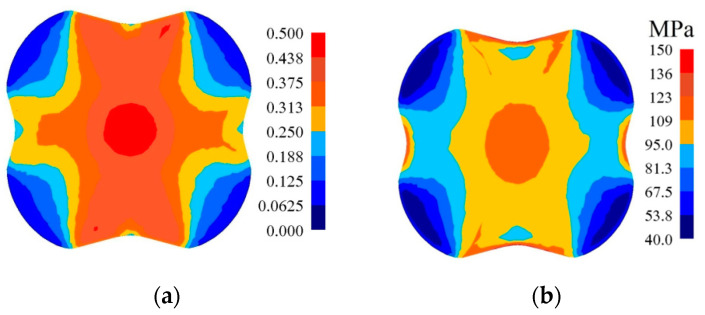

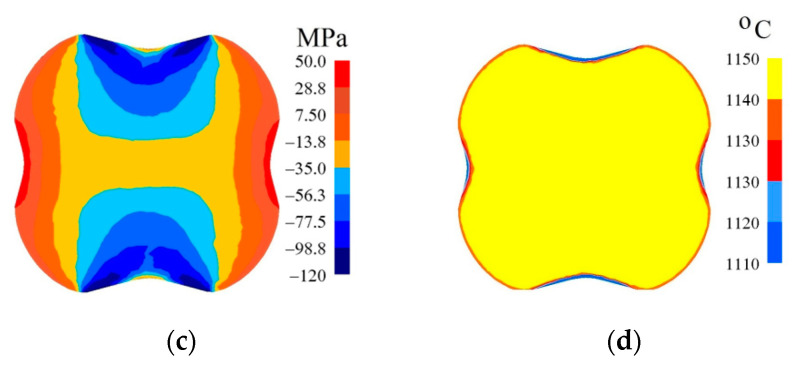
Distribution of the effective strain (**a**), effective stress (**b**), mean stresses (**c**), and temperature (**d**) on the cross-section of the workpiece in the course of forging in convex anvils after the second pass (*α_c_* = 150°, *ε_c_* = 0.22).

**Figure 9:**
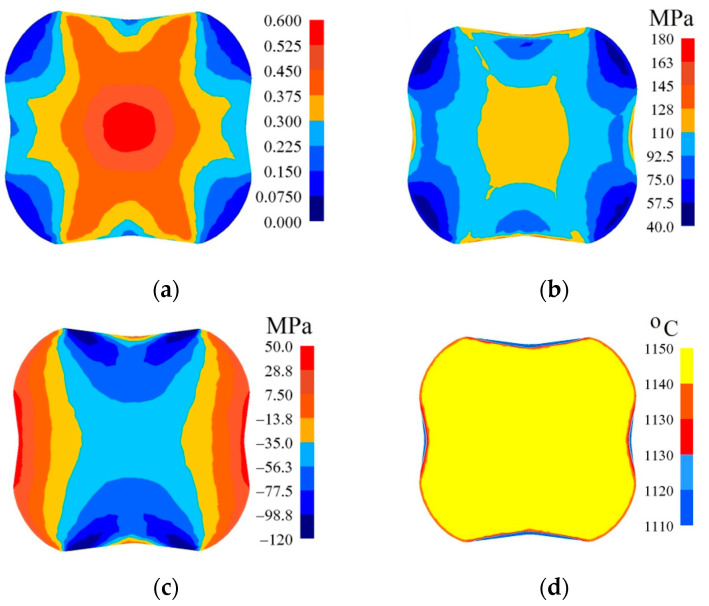
Distribution of the effective strain (**a**), effective stress (**b**), mean stresses (**c**), and temperature (**d**) on the cross-section of the workpiece in the course of forging in convex anvils after the second pass (*α_c_* = 165°, *ε_c_* = 0.22).

In Figure 7, Figure 8 and Figure 9, the distributions of the effective strain, the effective stress, mean stresses, and temperature after the second pass in convex anvils having the following angles: 135°, 150°, and 165°, in the case of the application of reduction ratio of *ε_c_* = 0.22, are presented. The values of the effective strain throughout most of the cross-section reached the following values: $\bar{\varepsilon }$ = 0.375–0.438 (135°, Figure 7a), $\bar{\varepsilon }$ = 0.438–0.500 (150°, Figure 8a) and $\bar{\varepsilon }$ = 0.525–0.600 (165°, Figure 9a). In the corner parts of the cross-section, the achieved values were significantly smaller in the case of the effective strain, and they amounted to, respectively, $\bar{\varepsilon }$ = 0.125–0.188, $\bar{\varepsilon }$ = 0.125–0.188, and $\bar{\varepsilon }$ = 0.150–0.225. A homogeneous distribution of the effective strain was accompanied by the distribution of the effective stress on the surface of the cross-section of a forging, which is presented in Figure 7b, Figure 8b and Figure 9b. The presented distribution of mean stresses in Figure 7c, Figure 8c and Figure 9c gives rise to the conclusive statement that it is exclusively on a small part of the lateral surface of specimens subjected to deformation that tensile stresses are possible to be observed. The distribution of temperature, presented in Figure 7d, Figure 8d and Figure 9d, confirms the stability of the surface of the cross-section of a forging; it was exclusively on contact surfaces between a hot metal and cooler anvils that a decrease in temperatures, amounting to Δ*T* = 40 °C, was observed.

**Figure 10:**
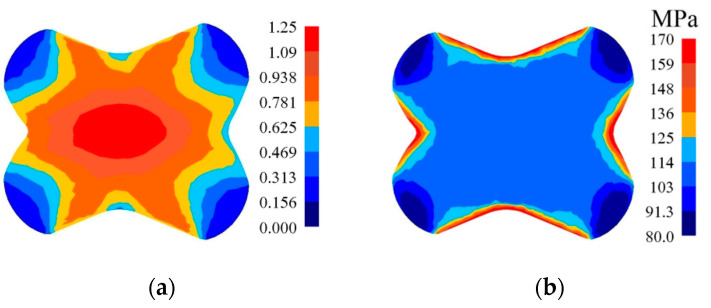

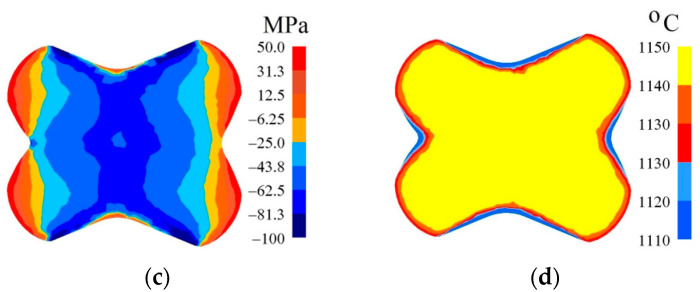
Distribution of the effective strain (**a**), effective stress (**b**), mean stresses (**c**), and temperature (**d**) on the cross-section of the workpiece in the course of forging in convex anvils after the second pass (*α_c_* = 135°, *ε_c_* = 0.51).

**Figure 11:**
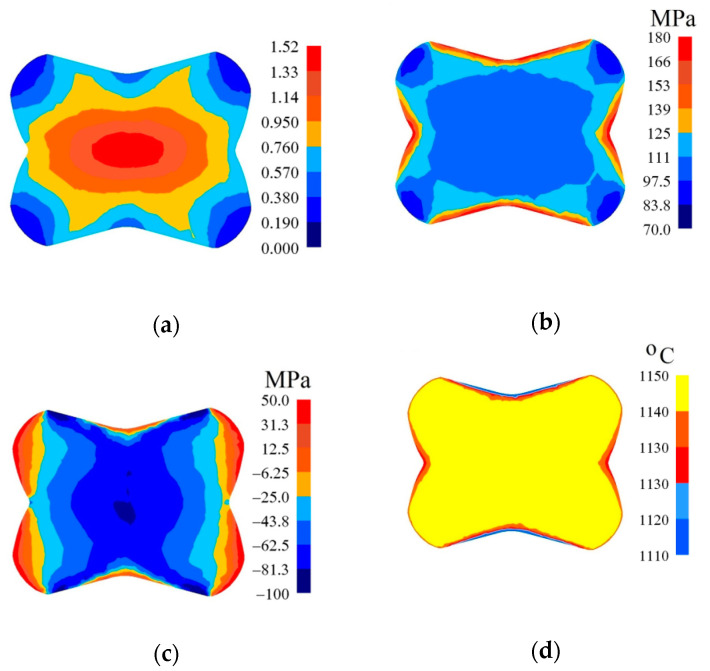
Distribution of the effective strain (**a**), effective stress (**b**), mean stresses (**c**), and temperature (**d**) on the cross-section of the workpiece in the course of forging in convex anvils after the second pass (*α_c_* = 150°, *ε_c_* = 0.51).

**Figure 12:**
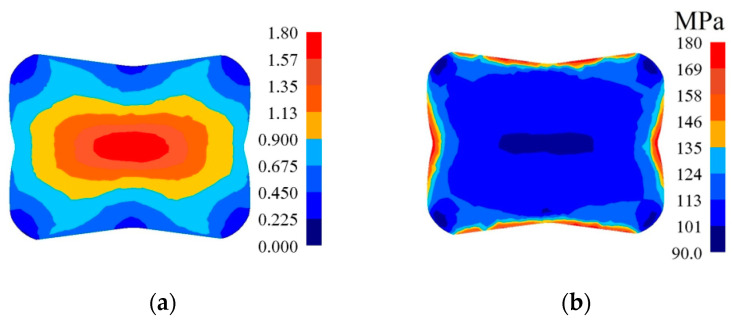

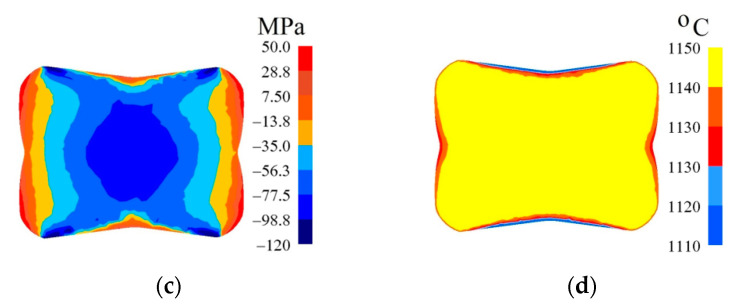
Distribution of the effective strain (**a**), effective stress (**b**), mean stresses (**c**), and temperature (**d**) on the cross-section of the workpiece in the course of forging in convex anvils after the second pass (*α_c_* = 165°, *ε_c_* = 0.51).

In Figure 10, Figure 11 and Figure 12, the distributions of the effective strain, the effective stress, mean stresses, and temperature after the second pass in convex anvils having the following angles: 135°, 150°, and 165°, in the case of the application of reduction ratio *ε_c_* = 0.51, are presented. A two-fold increase in the value of reduction ratio brought about a significant increase in the effective strain for the central part of the cross-section of a forging subjected to deformation in particular convex anvils, and it amounted to: $\bar{\varepsilon }$ = 1.09–1.25 (135°, Figure 10a), $\bar{\varepsilon }$ = 1.32–1.52 (150°, Figure 11a), and $\bar{\varepsilon }$ = 1.57–1.80 (165°, Figure 12a). In the corner parts of the cross-section, significantly smaller values of the effective strain, amounting to, respectively, $\bar{\varepsilon }$ = 0.313–0.469, $\bar{\varepsilon }$ = 0.380–0.570, and $\bar{\varepsilon }$ = 0.450–0.675, were achieved. A homogeneous distribution of the effective strain was accompanied by the distribution of the effective stress on the surfaces of the cross-section of forgings, and it amounted to $\bar{\sigma }$ = 111–114 MPa (Figure 10b, Figure 11b and Figure 12b). In the case of the presented distribution of mean stresses in Figure 10c, Figure 11c and Figure 12c, on a practically entire surface of the cross-section of a forging, favorable compression stresses were observed; it was exclusively on small areas situated in the corners of a forging that small values of tensile stresses were possible. A high value of the effective strain throughout most of the surface of the cross-section of a forging was conducive to generating significant values of the impact of plastic deformation converted into thermal energy, which was conducive to thermal stability in the internal layers of a forging (Figure 10d, Figure 11d and Figure 12d). It was exclusively on contact surfaces between a hot metal and cooler anvils that a decrease in temperatures, amounting to Δ*T* = 40 °C, was observed.

**Figure 13:**
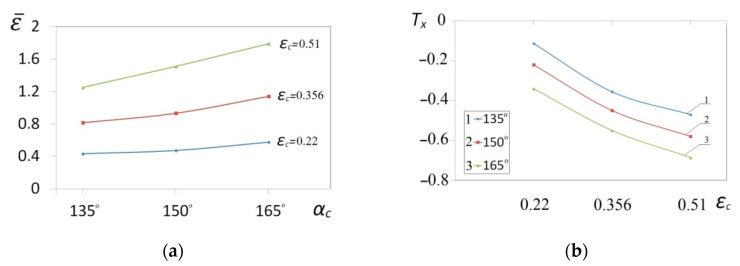
Influence of different angles of convex anvils *α_c_* and reduction ratio *ε_c_* on the effective strain $\bar{\varepsilon }$ (**a**) and the stress triaxiality ratio *T_x_* (**b**) in the axial zone.

The collation of the course of changes in the effective strain $\bar{\varepsilon }$ and stress triaxiality *T_x_* in the function of the shape of convex anvils, and also reduction ratio, is presented in Figure 13. The corner of a convex anvils, and also reduction ratio, demonstrated a significant influence upon the value of the effective strain in the axial zone of a forging, whereas an intensive increase in the effective strain was observed for convex anvils having the following angles: 150° and 165°, and also for the reduction ratio 0.356 and 0.51 (Figure 13a). In the case of convex anvils having the following angle: 165°, high values of strains were possible to be observed throughout the central part of a forging in the case of the reduction ratio 0.356 and 0.51, where the values of the effective strain were three times higher than a value of set strains, which was exerting a favorable influence upon reforging the axial zone, and which was conducive to eliminating a discontinuity formed by metallurgical means. In Figure 13b, the course of changes in stress triaxiality *T_x_* in the function of reduction ratio is presented for three investigations into convex anvils having the following angles: 135°, 150°, and 165°. A convex shape of working surfaces of convex anvils brought about the concentration of significant compression stresses in the central zone of a deformation valley in the case of negligibly small areas, not significant for forging technologies, and subjected to the impact of tensile stresses. Stress triaxiality, together with increase in reduction ratio, for particular applied convex anvils, reached larger and larger negative values, favorable for the stress state in a forging subjected to deformation. The most favorable conditions for reforging the axial zones of a forging were achieved in convex anvils having the following angle: 165°. 

### 3.3. Influence of the Shape of Concavities Cross-Section and Reduction Ratio on the Effective Strain and the Stressed State of the Workpiece during Cogging Process Variations Using Flat and V-Shaped Anvils

**Figure 14:**
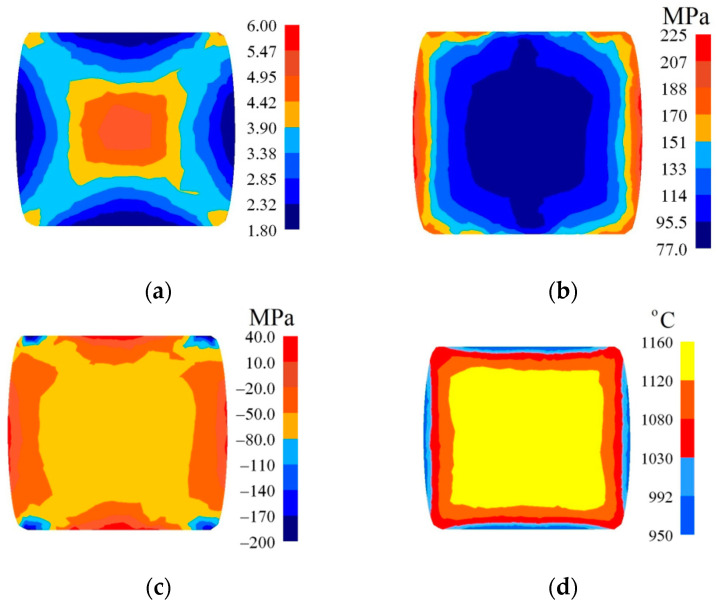
Distribution of the effective strain (**a**), effective stress (**b**), mean stresses (**c**), and temperature (**d**) after the eighth pass of the forging with a concave cross-section in flat anvils (*α_c_* = 150°, *ε_c_* = 0.356, forging ratio 4.2).

In Figure 14, the selected outcomes of investigations after eight passes, conducted into forgings with a concave cross-section (*α_c_* =150°, *ε_c_* = 0.356) in flat anvils, are presented. Those confirmed the occurrence of significant qualitative and quantitative changes in the distribution of the effective strain and effective stress, and also mean stresses. The largest values of the effective strain were observed in the central part of a forging, and they amounted to $\bar{\varepsilon }$ = 4.95–5.47 (Figure 14a). The area situated adjacently to the surfaces of anvils, and also the lateral zones of a forging, were exposed to the impact of the identical values of the effective strain, which were comparatively high, and which amounted to $\bar{\varepsilon }$= 2.85–3.37. The application of preliminary forging with the application of convex anvils brought about a significant increase in the values of the effective strain, and also about the homogeneous situation on the surface of the cross-section of a forging, in comparison with traditional forging in flat anvils [32]. Throughout most of the surface of the cross-section, the stability of the distribution of the effective stress was observed ($\bar{\sigma }$ = 95.5–114 MPa); it was exclusively on the lateral surfaces of a forging, as the result of decrease in the temperature, that the values of the effective stress were found to increase (Figure 14b). The distribution of mean stresses indicates the possibility of the occurrence of non-favorable tensile stresses exclusively on very small lateral surfaces of a forging, and is presented in Figure 14c. The forging process was accompanied by the stable distribution of temperature in the central part of a forging, brought about heat emission resulting from plastic deformation, and the contact surface between a hot metal and cool tools, and also lateral surfaces, were exposed to reduction in temperature, amounting to Δ*T =* 150–175 °C. 

**Figure 15:**
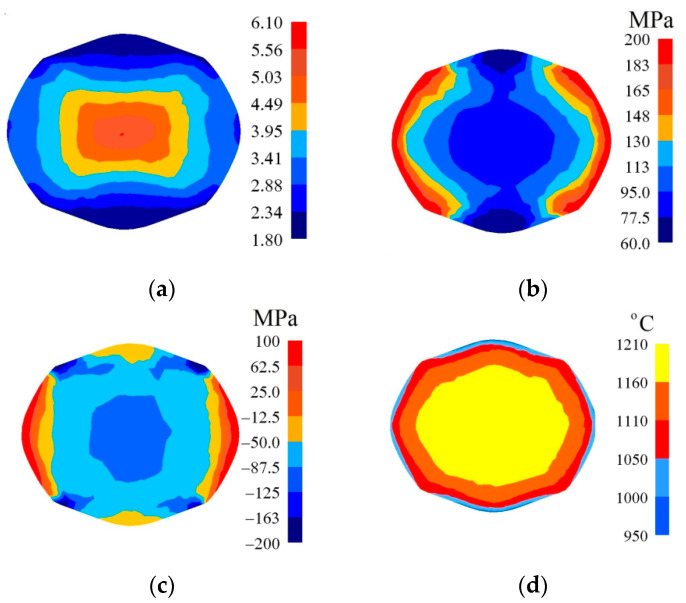
Distribution of the effective strain (**a**), effective stress (**b**), mean stresses (**c**), and temperature (**d**) after the fourth pass of the forging with a concave cross-section in V-shaped anvils (*α_c_* = 150°, *ε_c_* = 0.356, forging ratio 4.2).

In Figure 15, the distribution of the effective strain, the effective stress, mean stresses, and temperature after the fourth pass on a forging with a concave cross-section (*α_c_* = 150°, *ε_c_* = 0.356) in V-shaped anvils having the following impression angle: 140° × 140°, in the case of maintaining the identical forging ratio like in flat anvils (4.2), is presented. The largest values of the effective strain were achieved in the axial zone of a forging ($\bar{\varepsilon }$ = 5.56–6.10), which was exerting a favorable influence upon reforging this area (Figure 15a). The smallest values of the effective strain were achieved in the area below the vertexes of anvils ($\bar{\varepsilon }$ = 1.80–2.34); in the lateral zones of a forging, slightly larger values of the effective strain ($\bar{\varepsilon }$ = 2.87–3.41) were achieved. The similar distribution of the effective stress is presented in Figure 15b. Throughout most of the surface of the cross-section, the stability of the distribution of the effective stress was observed ($\bar{\sigma }$ = 95–113 MPa); it was exclusively on the lateral surfaces of a forging, as the result of decrease in the temperature, that the values of the effective stress were found to increase. In the course of forging in these anvils, no tensile stresses were ascertained in the axial zone of a forging. The distribution of mean stresses presented (Figure 15c) indicates the possibility of the occurrence of tensile stresses exclusively on a very small area situated in the lateral zones of a forging. The forging process was accompanied by a stability distribution of temperature throughout most of the cross-section of a forging, brought about heat emission resulting from plastic deformation and the work of friction forces (Figure 15d). On a contact surface between a metal and anvils, and also on the lateral surfaces of a forging, a decrease in temperature amounted to Δ*T =* 100–130 °C.

**Figure 16:**
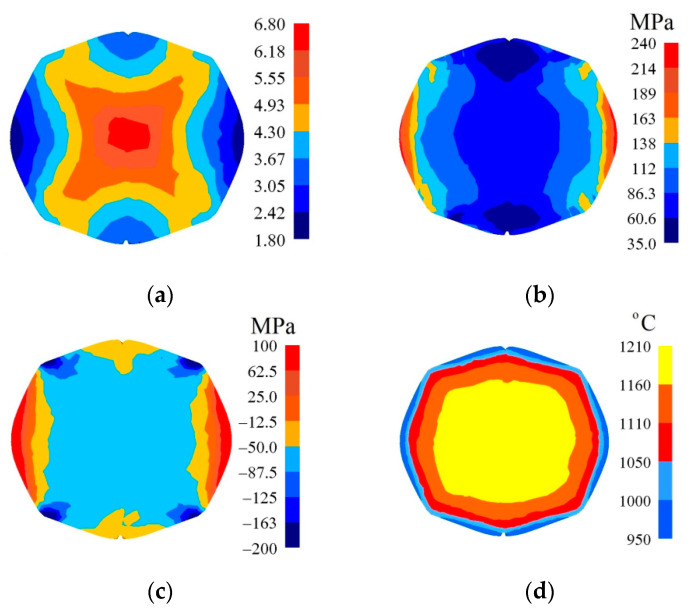
Distribution of the effective strain (**a**), effective stress (**b**), mean stresses (**c**), and temperature (**d**) after the fourth pass of the forging with a concave cross-section in V-shaped anvils (*α_c_* = 165°, *ε_c_* = 0.51, forging ratio 4.2).

In Figure 16, the distribution of the effective strain, the effective stress, mean stresses, and temperature after the fourth pass on a forging with a concave cross-section (*α_c_* = 165°, *ε_c_* = 0.51) in V-shaped anvils having the following impression angle: 140° × 140°, in the case of maintaining the identical forging ratio like in flat anvils (4.2), is presented. The application of preliminary forging with the application of convex anvils having a larger angle and higher reduction ratio brought about a significant increase in the effective strain in the axial zone of a forging reaching the value of $\bar{\varepsilon }$ = 6.18–6.80 (Figure 16a), contributing to a good reforging of this area and eliminating the remnants of metallurgical defects. In remaining zones of a forging, the achieved values of the effective strain were similar to those achieved in the previous case. Throughout most of the cross-section, a homogeneous distribution of the effective stress ($\bar{\sigma }$ = 86.3–112 MPa) was achieved; it was exclusively on the lateral surfaces of a forging, as the result of a decrease in the temperature, that the values of the effective stress were found to increase (Figure 16b). The presented distribution of mean stresses (Figure 16c) indicates the possibility of the occurrence of tensile stresses exclusively on a very small area situated in the lateral zones of a forging. The forging process was accompanied by a stability distribution of temperature throughout most of the cross-section of a forging, brought about heat emission resulting from plastic deformation and the work of friction forces (Figure 16d). On the contact surface between a metal subjected to deformation and anvils, and also on the lateral surfaces of a forging, a decrease in temperature amounted to Δ*T =* 100–130 °C. 

**Figure 17:**
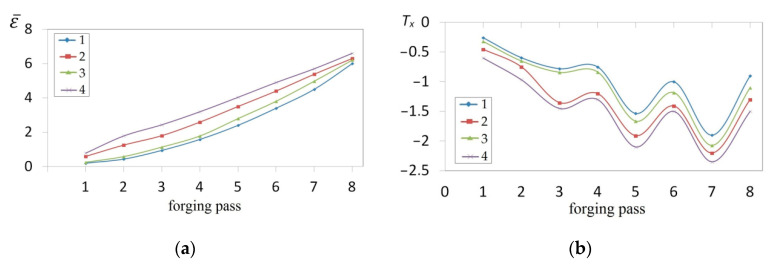
Influence of different passes upon the effective strain $\bar{\varepsilon }$ (**a**) and the stress triaxiality ratio Tx (b) in the axial zone in the course of the cogging in flat anvils of the forging with a concave cross-section for different angles and reduction ratio: 1—*ε_c_* = 0.22, *α_c_* = 135°; 2—*ε_c_* = 0.51, *α_c_* = 135°; 3—*ε_c_* = 0.22, *α_c_* = 165°; 4—*ε_c_* = 0.51, *α_c_* = 165°.

The collation of the courses of changes in the effective strain $\bar{\varepsilon }$ and stress triaxiality *T_x_* for particular passes in the case of forgings with selected concavities and subjected to deformations in flat anvils is presented in Figure 17. In the further passes, an increase in the total effective strain was observed in the case of a more favorable stress state in a forging subjected to deformation. The angle of the concavity achieved by means of deformation in convex anvils, and also reduction ratio, demonstrated a significant influence exerted upon values of the effective strain in the axial zone of a forging subjected to deformation in flat anvils. The highest increase in the effective strain was observed for concavities achieved in convex anvils having the following angle: 165°, and for reduction ratio *ε_c_* = 0.51 (Figure 17a). For the reduction ratio *ε_c_* = 0.22 in particular passes, significantly smaller values of the effective strain were achieved, determined, in addition to that, by the shape of concavities. In Figure 17b, the courses in changes in stress triaxiality *T_x_* for particular passes in the case of forgings having selected concavities with the following angle: 135° and 165°, and values reduction ratio: *ε_c_* = 0.22 and *ε_c_* = 0.51 forged in flat anvils, is presented. Stress triaxiality, together with increase in reduction ratio and concavity angle (*ε_c_* = 0.51 and *α_c_* = 165°), assumed larger negative values, favorable for the stress state in a forging subjected to deformation. For those parameters of deformation conducted in flat anvils, the most favorable conditions for reforging the axial zones of a forging were achieved.

**Figure 18:**
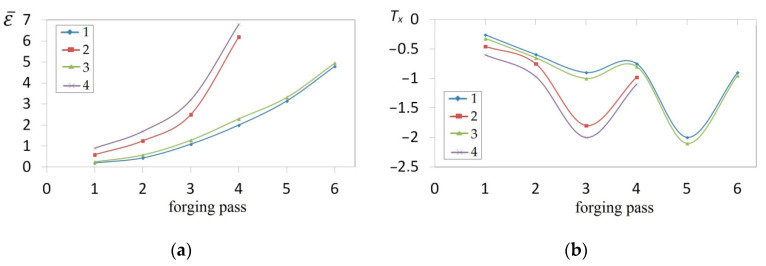
Influence of different passes upon the effective strain $\bar{\varepsilon }$ (**a**) and the stress triaxiality ratio *T_x_* (**b**) in the axial zone in the course of the cogging in V-shaped anvils of the forging with a concave cross-section for different angles and reduction ratio: 1—*ε_c_* = 0.22, *α_c_* = 135°; 2—*ε_c_* = 0.51, *α_c_* = 135°; 3—*ε_c_* = 0.22, *α_c_* = 165°; 4—*ε_c_* = 0.51, *α_c_* = 165°.

The collation of the course of changes in the effective strain $\bar{\varepsilon }$ and stress triaxiality *T_x_* for particular passes in the case of forgings with selected concavities, subjected to deformation in V-shaped anvils, is presented in Figure 18. In the further passes, an increase in the total effective strain, in the case of more favorable stress state in a forging subjected to deformation, was found to occur. The shape of a concavity achieved by means of shaping in convex anvils, and also the applied reduction ratio, demonstrated as well a significant influence exerted upon the values of the effective strain in the central part of a forging subjected to deformation in V-shaped anvils. Like in the case of flat anvils, the highest increase in the effective strain was observed for concavities achieved in convex anvils having the following angle: 165°, and also for reduction ratio: *ε_c_* = 0.51 ($\bar{\varepsilon }$ = 6.80), achieved in the fourth pass. A required quantity of passes in shaped anvils was smaller by half in comparison with shaping in flat anvils (Figure 18a). For the concavity achieved by means of the following reduction ratio: *ε_c_* = 0.22, the required number of passes was found to increase (six passes), and significantly smaller values of the effective strain were achieved in that case; in addition to that, the shape of concavity is determined. In Figure 18b, the course of changes in stress triaxiality *T_x_* for particular passes in the case of forgings with selected concavities having the following angle: 135° and 165°, and applied reduction ratio *ε_c_* = 0.22 and *ε_c_* = 0.51 forged in V-shaped anvils, is presented. Stress triaxiality, together with increase in reduction ratio and the angle of concavities (*ε_c_* = 0.51 and *α_c_* = 165°), similarly to what occurs in flat anvils, assumed favorably negative values. A convex shape of the working surfaces of anvils having the following angle: 165° and a high value of reduction ratio brought about, at an initial stage of deformation, concentrating a significant compression of stresses in the central part of a deformation valley, in the case of negligibly small areas subjected to the impact of tensile stresses. The further forging process conducted in V-shaped anvils contributed to an increase in the concentration of compression stresses in the volume of a forging, and also to eliminating non-favorable tensile stresses situated in the lateral zones of a shaped material.

### 3.4. Results Prediction for Several Ductile Fracture Criteria

**Figure 19:**
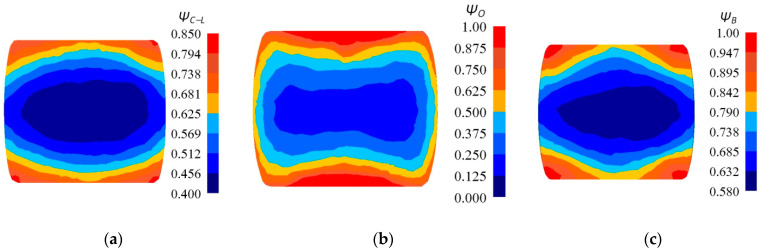
Distribution of the damage factors: (**a**) Cockcroft and Latham, (**b**) Oyane et al., and (**c**) Brozzo et al. after eighth pass of the forging with a concave cross-section in flat anvils (α_c_ = 150°, ε_c_ = 0.356, forging ratio 4.2).

In Figure 19, the distribution of the damage factors for the three selected criteria of cracking after the eighth pass of the forging with a concave cross-section in flat anvils (the parameters of the concavities: *α_c_* =150°, *ε_c_* = 0.356), is presented. What was conducted, was the calculation of the value of selected criteria of cracking, and it rendered it possible to determine the areas exposed to the greatest danger of crack formation. In the axial zone of a forging, the smallest values of crack formation factor were achieved for Oyane et al. criterion (*Ψ_O_* = 0.250–0.375), whereas the largest values for Brozzo et al. criterion (*Ψ_B_* = 0.632–0.685). The intermediate values were achieved in the case of the application of the normalized Cockcroft and Latham criterion (*Ψ_C-L_* = 0.456–0.513). In the case of assuming the normalized Cockcroft and Latham criterion as the basic one, the one by Oyane et al. decreases the values of crack formation factor in the axial zone of a forging by 35.5%, whereas for this zone the criterion of Brozzo et al. gives rise to expecting the values of the crack formation factor increased by 35.9%. Numerical calculation conducted in the case of the application of these criteria demonstrated the same areas where the values of crack formation factors reached or exceeded the determined boundary values for particular criteria. That means that, in a material subjected to deformation, the maximum possible level of plasticity was reached, and the formation of discontinuity is possible. The largest values of the crack formation factors were achieved in the corners of a forging subjected to deformation, and it is also there that positive values of mean stresses are observed (Figure 14c). These are tensile stresses, which are conducive to crack formation in these areas, the largest values of the effective stress are concentrated (Figure 14b), and also the corners of a forging subjected to deformation have the lowest temperature (Figure 14d). These areas are known from industrial practice as the most frequent situations of crack formation in the course of forging in flat anvils.

**Figure 20:**
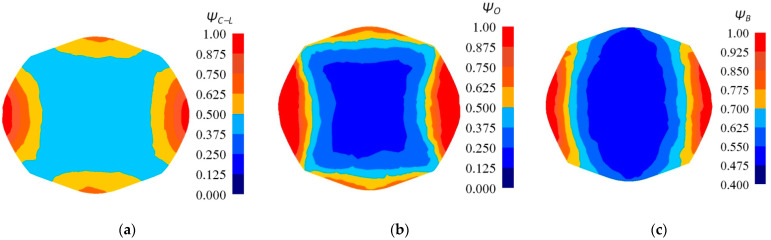
Distribution of the damage factors: (**a**) Cockcroft and Latham, (**b**) Oyane et al., and (**c**) Brozzo et al. after fourth pass of the forging with a concave cross-section in V-shaped anvils (α_c_ = 150°, ε_c_ = 0.356, forging ratio 4.2).

In Figure 20, the distribution of the damage factors for the three selected criteria of cracking after the fourth pass of the forging with a concave cross-section in V-shaped anvils (the parameters of the concavity: *α_c_* = 150°, *ε_c_* = 0.356) is presented. In the axial zone of a forging, the smallest values of the crack formation factor were achieved for the criterion of Oyane et al. (*Ψ_O_* = 0.250–0.375), whereas the highest ones for Brozzo et al. criterion (*Ψ_B_* = 0.550–0.625). The intermediate values were achieved in the case of the application of the normalized Cockcroft and Latham criterion (*Ψ_C-L_* = 0.500). Assuming the normalized Cockcroft and Latham criterion as a basis, it is ascertained that Oyane et al. criterion decreases the values of the crack formation factor in the axial zone of a forging by 37.6%, whereas for the same zone, the criterion of Brozzo et al. gives rise to expecting the larger values of the crack formation factor (the difference amounts to 17.5%). Numerical calculations conducted in the case of the application of these criteria indicated, in the course of forging in V-shaped anvils, these same areas on the lateral surfaces of forgings, where the values of the crack formation factors reached, or to a non-significant degree, exceeded the determined boundary values for particular criteria. That means that, in a material subjected to deformation, the maximum possible level of plasticity was reached, and that initiating the formation of discontinuity is possible. The largest values of the crack formation factors were achieved in small areas situated on the lateral surfaces of forgings subjected to deformation, and it was there as well that positive values of mean stresses are observed (Figure 15c). These are tensile stresses, which are conducive to crack formation. In these areas, the largest values of the effective stress are concentrated (Figure 15b), and also the corners of a forging subjected to deformation have the lowest temperature (Figure 15d). In these small areas, crack formation is possible in the course of V-shaped anvils.

**Figure 21:**
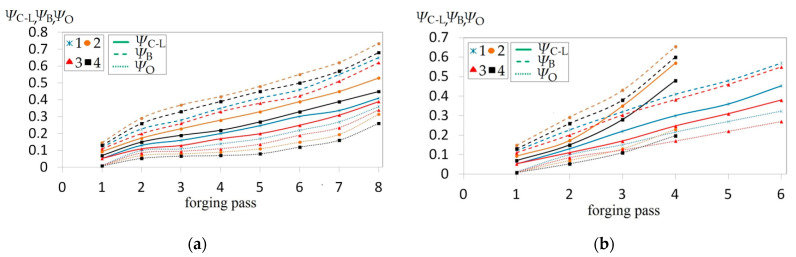
Influence exerted by of different passes upon the damage factors: Cockcroft and Latham (*Ψ_C-L_*), Oyane et al. (*Ψ_O_*), and Brozzo et al. (*Ψ_B_*) in the axial zone in the course of cogging of the forging with a concave cross-section in flat anvils (**a**) and in V-shaped anvils (**b**) for different angles and reduction ratio: 1—*ε_c_* = 0.22, *α_c_* = 135°; 2—*ε_c_* = 0.51, *α_c_* = 135°; 3—*ε_c_* = 0.22, *α_c_* = 165°; 4—*ε_c_* = 0.51, *α_c_* = 165°.

The collation of the course of changes in the crack formation factors for the axial zone of a forging in accordance with three analyzed criteria, and also for particular passes with selected concavities having the following parameters: *α_c_* = 135° and *α_c_* = 165°, *ε_c_* = 0.22 and *ε_c_* = 0.51, subjected to deformation in flat anvils, is presented in Figure 21a. In the further passes, an increase in total values of the particular crack formation factors was observed, whereas the largest values were achieved for Brozzo et al. criterion, and the lowest ones for Oyane et al. criterion. The intermediate values were achieved in the case of the application of the normalized Cockcroft and Latham criterion. The shape of concavity achieved by means of deformation in convex anvils and a certain angle *α_c_*, and also reduction ratio *ε_c_*, demonstrated a significant influence exerted upon the values of particular criteria in the axial zone of a forging subjected to deformation in flat anvils. For a concavity having the following angle: *α_c_* = 165°, more favorable, smaller values of the particular criteria than for *α_c_* = 135°, and the same correlation was observed to occur for each and every value of height of the concavity of a certain reduction ratio *ε_c_*. For height of concavity of a certain reduction ratio *ε_c_* = 0.22, achieved in particular passes significantly smaller values of particular analyzed criteria of crack formation. Increase in the height of the concavity of a certain reduction ratio *ε_c_* = 0.51 brought about increase in the analyzed criteria of crack formation in the further passes, and also for particular angles of concavity. In the axial zone of a forging, none of the analyzed criteria reached the boundary values.

The collation of the course of changes in the crack formation factors for the axial zone of a forging in accordance with three analyzed criteria, and also for particular passes with selected concavities having the following parameters: *α_c_* = 135° and *α_c_* = 165°, *ε_c_* = 0.22 and *ε_c_* = 0.51, subjected to deformation in V-shaped anvils, is presented in Figure 21b. The largest values were achieved for Brozzo et al. criterion, whereas the lowest ones for Oyane et al. criterion. The intermediate values were achieved in the case of the application of the normalized Cockcroft and Latham criterion. The shape of concavity achieved by means of deformation in convex anvils and a certain angle α_c_, and reduction ratio ε_c_ demonstrated a significant influence exerted upon the values of particular criteria in the axial zone of a forging subjected to deformation in V-shaped anvils. For a concavity having the following angle: *α_c_* = 165° more favorable, smaller values of particular criteria than for *α_c_* = 135° were achieved, and the same correlation was observed to occur for each and every value of height of the concavity of a certain reduction ratio *ε_c_*. Increase in the height of concavity of a certain reduction ratio *ε_c_* = 0.51 brought about a significant increase in the analyzed criteria, particularly in the fourth (last) pass. For concavities, the achieved reduction ratio *ε_c_* = 0.22, a required number of passes was increased to 6, in which significantly smaller values of the particular analyzed criteria crack formation and their slow increase were achieved. In the axial zone of a forging subjected to deformation in V-shaped anvils, none of the analyzed criteria reached the boundary values.

### 3.5. Experimental Verifications of the Simulation Model 

**Figure 22:**
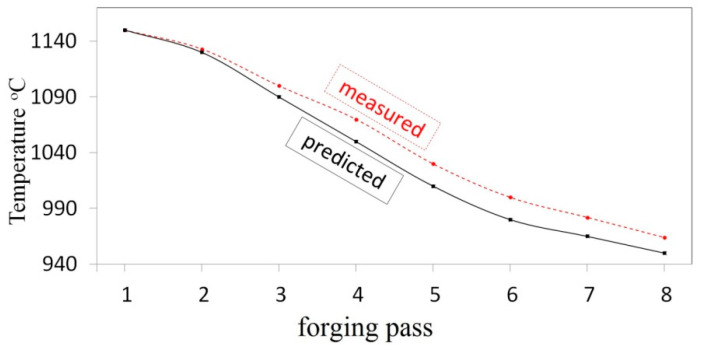
Comparison of the computed and the measured temperature during the cogging process of the X5CrNi18-10 steel forged in flat anvils, forging ratio 4.2.

The comparison of calculated and measured temperature in selected points on the deformed surface of a forging after particular passes is presented in Figure 22. The measurement of temperature at selected points on the surface of a deformed cogging, after particular technological passes, was conducted with the application of a thermal-visual camera. What was conducted, was the comparative analysis of temperature according to numerical calculations and achieved by means of experiments; they demonstrated a comparatively good level of being commensurate.

**Figure 23:**
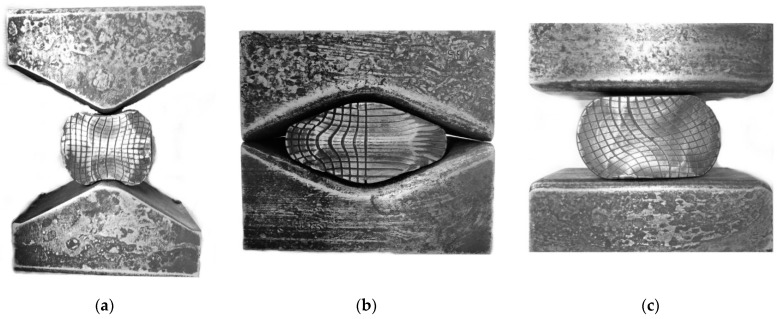
Shape of the anvils used in the laboratory experimentation: (**a**) convex (*α_c_* = 135°), (**b**) V-shaped (*α* = 140° × 140°), and (**c**) flat.

In Figure 23, the shapes of anvils applied in the experimental investigations are presented: convex (*α_c_* = 135°), V-shaped (*α* = 140° × 140°), and flat.

**Figure 24:**
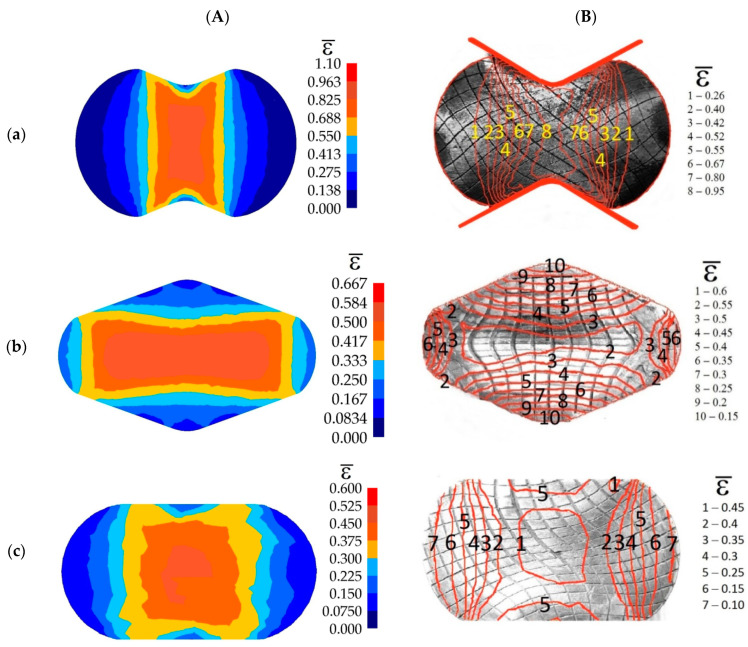
Comparisons of the numerical (**A**) and experimental (**B**) distribution of effective strain on the cross-section surfaces of the X5CrNi18-10 steel specimens deformed in anvils: (**a**) convex (*α_c_* = 135°, *ε_c_* = 0.693), (**b**) V-shaped (*α* = 140° × 140°, *ε_h_* = 0.35), and (**c**) flat (*ε_h_* = 0.35).

Experimental confirmation of the outcomes of investigations into numerical distribution of the effective strain after forging in convex, V-shaped, and flat anvils is presented in Figure 24. The state of strain was determined with the application of the coordination grid method. The experimental investigations were conducted on cylindrical specimens having the following dimensions *φ*80 × 150 mm and made of the X5CrNi18-10 steel. In the division plane, a square coordination grid (3.0 × 3.0 mm^2^) was plotted with the precision of 0.01 mm. The situation of process units before and deforming was measured on a measuring microscope coupled with a computerized workstation. The specimens were subjected to deformation on a hydraulic press (pressure: 500 kN). For determining the components of the Cauchy strain tensors, the relocation field of a sixth-degree double-parameter polynomials was approximated, whereas coefficients were determined with the application of the method of least squares, taking under consideration the condition of a constant volume (Lagrange multiplier method). The comparative analysis of effective strain based upon numerical calculations, and obtained by means of experiments, demonstrated their being mutually commensurate to a relatively high degree.

## 4. Conclusions

Numerical investigations, confirmed by selected experimental research, rendered it possible to determine the local values typical in the case of the strain state, stress state, the distribution of temperature, and also the possibility of cracking a material in the form of three selected ductile fracture criteria: the normalized Cockcroft and Latham criterion, Oyane et al. criterion, and Brozzo et al. criterion. In the presented paper, the focus of attention was upon the analysis of a new, two-stage manner of cogging of a forging-type shaft consisting in preliminary shaping of an ingot in convex anvils having convex working surfaces, and also principal forging conducted in flat or shaped anvils. The new manner of forging brought about the formation of favorable conditions for achieving the maximum values of the effective strain in the axial zone of a forging, accompanied by a simultaneous absence of tensile stresses, which was improving reforging and quality of achieved forgings. What was determined, was the effective geometric shapes of convex anvils; the efficiency of different technological parameters in the case of intensity of reforging the axial zone of an ingot was also analyzed. It was recommended to conduct preliminary shaping in convex anvils having the following angle: 165°, and the depth of achieved concavities ought to be matching the reduction ratio of *ε_c_* ≥ 0.356. Taking under consideration the achieved values of the effective strain in the axial zone of a forging and the distributions of mean stresses, the further forging process can be conducted in flat anvils, or in the V-shaped ones. 

The course of changes in certain values of particular criteria in the course of forging in different investigated anvils, with different values reduction ratios, informed about the values of criteria in particular zones of plastic deformation, and rendered it possible to the situations and phases of deformation, in which the loss of cohesion of a material subjected to deformation is possible. In further passes, an increase in the total values of the particular crack formation factors was observed, whereas the largest values were achieved for Brozzo et al. criterion, and the lowest ones for Oyane et al. criterion. The intermediate values were achieved in the case of the application of the normalized Cockcroft and Latham criterion. The shape of concavity achieved by means of deformation in convex anvils and a certain angle *α_c_*, and also reduction ratio *ε_c_*, demonstrated significant influence exerted upon the values of particular criteria in the axial zone of a forging. In the axial zone of a forging for the forging ratio amounting to 4.2, none of the analyzed criteria reached the boundary values.

## Figures and Tables

**Table 1:** Schedule of the cogging process.

Shape of Anvils	Convex Anvils *α_c_*	Reduction Ratio *ε_c_*	Number of Passes: H-Height and S-Side of Square, mm							
			1	2	3	4	5	6	7	8
Flat			H_1_	H_1_	H_1_	H_1_	H_2_	S_1_	H_3_	S_2_
	135°	0.22	640	640	640	640	448	490	294	345.5
	150°	0.36	560	560	560	560	399	430	314	345.5
	165°	0.51	480	480	480	480	343	370	334	345.5
V-shaped			H_1_	H_1_	H_2_	H_2_	H_3_	H_3_		
	135°	0.22	640	640	590	590	405	405		
	150°	0.36	560	560	405	405				
	165°	0.51	480	480	405	405				

## Data Availability

The author confirms that the data supporting the findings of this study are available within the article.
